# ROS-responsive biomimetic nanoparticles for potential application in targeted anti-atherosclerosis

**DOI:** 10.1093/rb/rbab033

**Published:** 2021-07-18

**Authors:** Dan Tang, Yi Wang, Andy Wijaya, Boyan Liu, Ali Maruf, Jinxuan Wang, Jianxiong Xu, Xiaoling Liao, Wei Wu, Guixue Wang

**Affiliations:** 1 Key Laboratory for Biorheological Science and Technology of Ministry of Education, State and Local Joint Engineering Laboratory for Vascular Implants, Bioengineering College of Chongqing University, Chongqing 400030, China; 2 Chongqing Key Laboratory of Nano/Micro Composite Material and Device, School of Metallurgy and Materials Engineering, Chongqing University of Science and Technology, Chongqing 401331, China

**Keywords:** biomimetic nanoparticles, macrophage membrane, ROS-responsive, targeted delivery, atherosclerosis

## Abstract

The development of nanomedicines provides new opportunities for the treatment of atherosclerosis (AS) due to their great advantages such as the improved drug solubility, enhanced bioavailability and reduced side effects. Despite these advantages, nanomedicines are still facing some challenges. The problems remain in the short circulation life, lack of specific targeting and poor drug release controllability. In order to overcome the shortages of conventional nanomedicines, the combination of biomimetic strategy with smart nanoagents has been proposed. In light with the high reactive oxygen species (ROS) level in AS microenvironment and the fact that macrophages play a critical role in the pathogenesis of AS, we fabricated ROS-responsive biomimetic nanoparticles (NPs), which camouflaged macrophage membrane (MM) on ROS-responsive NPs loaded with rapamycin (RNPs) for potential application in AS therapy. The resulting ROS-responsive biomimetic NPs (MM/RNPs) exhibited favorable hydrodynamic size with negative surface charge, retained the functional proteins from MM, and showed ROS-responsive drug release. Because of the biomimetic camouflaging on surface, MM/RNPs could effectively escape from macrophages uptake and target to inflammatory endothelial cells. Meanwhile, MM/RNPs could inhibit the proliferation of macrophages and smooth muscle cells *in vitro*. Furthermore, the MM-coated NPs were found to be nontoxic in both cytotoxicity assay and *in vivo* toxicity evaluation. Consequently, these results demonstrated that MM/RNPs could be a potential candidate of drug delivery system for safe and effective anti-AS applications.

## Introduction

Cardiovascular disease is a general term for cardiovascular and cerebrovascular diseases. It is one of the leading causes to death worldwide because of its high morbidity and mortality [1]. Atherosclerosis (AS), as a typical chronic inflammatory vascular disease, is the main pathological contributor underlying cardiovascular disease [2, 3]. Recently, oral lipid-lowering drugs are the most used clinical practices to prevent AS. However, these orally administered drugs showed limited therapeutic outcome due to their nonspecific distribution, serious side effects and the rapid clearance by organisms [4, 5]. In recent years, drug delivery system (DDS) has demonstrated exciting possibilities for diagnosis and treatment of AS [6−8]. DDS can prolong the drug half-life, improve site-specific targeting and reduce side effects of the molecule drugs, which meets the demands of new strategies for AS therapy.

However, the clinical application of the synthetic DDS is extremely limited because of the existence of various biological barriers [9−11]. For instance, the intravenously administered nanomedicine may be endocytosed by mononuclear phagocyte system [12]. Therefore, it is imperative to design advanced nanocarriers to overcome the obstacles of biological barriers. Recently, biomimetic camouflage strategy has attracted special attention [13−15]. By fussing natural cell membranes onto synthetic cores, the cell membrane-camouflaged nanoparticles (NPs) are biocompatible, biodegradable and non-immunogenic. Through this approach, the biomimetic NPs have benefits for prolonging the blood circulation time, reducing the undesirable clearance, and enhancing the specific targeting ability [16−18]. Inspired by the cell membrane coating strategy, the construction of an intelligent biomimetic DDS could be an effective approach for the targeted therapy of AS.

Studies have revealed that AS is initiated by the damaging of endothelium [19, 20]. Subsequently, the dysfunctional endothelial cells continuously secrete a series of adhesion molecules, which are responsible for the recruitment of immune cells. Therefore, inflammatory endothelial cells are ideal pathological targets for the design of DDS to treat AS. In addition, in the process of AS, macrophages are recruited to the lesion site and participate in all stages of the development of AS [21, 22]. Meanwhile, as a kind of immune cells, macrophages have innate immune escape function [23, 24]. Therefore, coating macrophage membrane (MM) onto the surface of NPs is a potential strategy to achieve long-term circulation and specific targeting to AS.

In our previous work [25], MM camouflaged PLGA NPs have been fabricated for targeted AS treatment. The results showed that the coated NPs could inhibit macrophage-mediated phagocytosis, prolong blood circulation and delay plaque progression, which confirmed the great advantages of applying MM in biomimetic nanomedicine for AS therapy. However, PLGA NPs are facing the challenge of slow drug release at the disease lesion site, resulting in limited therapeutic effect. Therefore, we sought to further develop an MM-coated stimulus-responsive platform with intelligent drug release ability and better safety profile as the continuation and amelioration of the previous work.

Strong evidence has indicated that the microenvironment of AS lesion site is rich in ROS [26, 27]. Accordingly, applying ROS-responsive NPs in AS therapy represents an intriguing approach. According to previous reports, boronic esters can be used as oxidation-labile groups in response to ROS stimulus, which could contribute to the controllable drug release at the disease site in the presence and absence of ROS [28, 29]. The strategy of using boronic ester modified polymers to construct ROS-responsive nanomedicines for the management of AS have great potential of achieving the desirable therapeutic benefits [30, 31].

**Figure 1.**
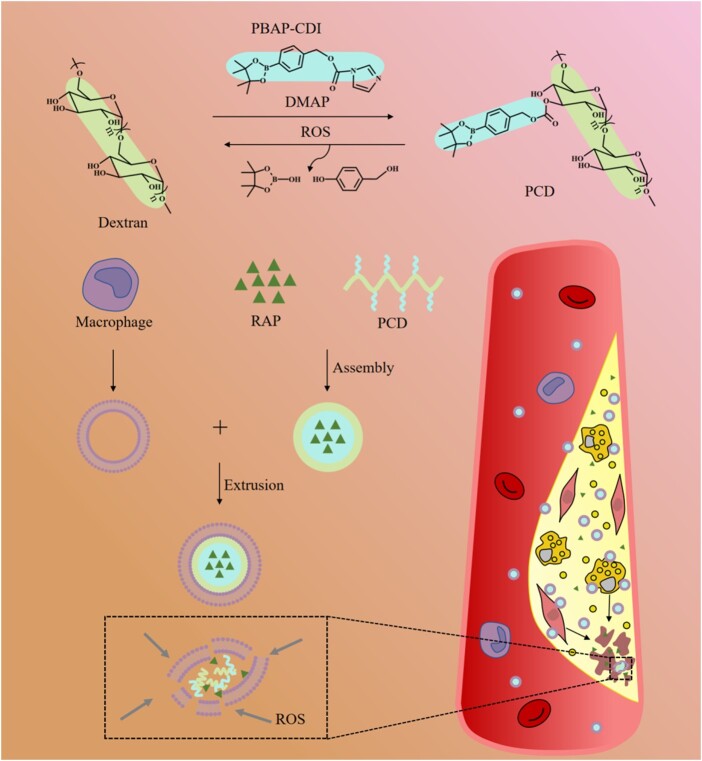
Illustration of MM/RNPs delivering and releasing RAP into atherosclerotic plaques, enhancing the therapeutic effect. PBAP, 4-phenylboronic acid pinacol ester; CDI, carbonyldiimidazole; DMAP, 4-dimethylaminopyridine; ROS, reactive oxygen species; RAP, rapamycin; MM, macrophage membrane; MV, macrophage vesicle.

Herein, we sought to fabricate an ROS-responsive biomimetic nanoplatform for targeted AS treatment. It is well known that AS is a chronic inflammatory disease, therefore, anti-inflammatory drugs may have a considerable potential to manage AS [32, 33]. In this work, rapamycin (RAP) was used as a model drug due to its exhibition of effective anti-inflammatory function. The boronic ester modified dextran, which is biodegradable and biocompatible, was used as a drug carrier. MMs were coated on the surface of NPs to obtain the MM-coated ROS-responsive NPs for enhancing the targeted therapy of AS (Fig. 1).

## Materials and methods

### Materials

4-(Hydroxymethyl) phenylboronic acid pinacol ester (PBAP), dextran with molecular weight (MW) of 5000 g/mol, and ferrous perchlorate were purchased from Shanghai Aladdin Biochemical Technology Co., Ltd (Shanghai, China). 4-Dimethylaminopyridine (DMAP) and NaClO were purchased from Shanghai Macklin Biochemical Co., Ltd (Shanghai, China). N,N’-Carbonyldiimidazole (CDI) was purchased from J&K Chemical Co., Ltd. (Shanghai, China). RAP was purchased from Dalian Meilun Biotechnology Co., Ltd (Dalian, China). Deuterated dimethyl sulfoxide (DMSO-*d_6_*) and deuterated methanol (methanol-*d_4_*) were purchased from Sun Chemical Technology Co., Ltd (Shanghai, China). Hydrogen peroxide (H_2_O_2_) was purchased from ChengDu Chron Chemicals Co., Ltd (Chengdu, China). DAPI and cell total protein extraction kits were purchased from Beyotime Institute of Biotechnology Co., Ltd (Jiangsu, China). 1,19-Dioctadecyl-3,3,39,39-tetramethylindodicarbocyanine perchlorate (DiD) was purchased from Biotium Inc. (Fremont, USA). The CellTiter 96^TM^ AQueous One Solution Cell Proliferation Assay (MTS) was purchased from Thermo Fisher Scientific (SanJose, CA, USA).

### Synthesis of PBAP-CDI and PCD

The activation of PBAP was performed in the presence of excess amount of CDI. Briefly, 649 mg of CDI (4 mmol) was dissolved in 6 ml of anhydrous CH_2_Cl_2_, into which 468 mg of PBAP (2 mmol) was added and then kept stirring for 1 h under 30°C. The mixture was thoroughly washed with deionized water and saline, evaporated and vacuum-dried to obtain solid activated product (PBAP-CDI). Then, the ROS-responsive polymer (PCD) was synthesized by conjugating boronic ester onto dextran. It was carried out by an esterification reaction between dextran-OH and PBAP-CDI using DMAP as a coupling catalytic agent. Specifically, 45 mg of dextran (MW 5000 g/mol, 0.009 mmol) was dissolved in 5 ml of anhydrous DMSO, into which 180 mg of PBAP-CDI (0.55 mmol) was added. Reaction was carried out at 37°C under magnetic stirring overnight. The collected product was dialyzed using a dialysis bag (MW cut-off (MWCO) of 3500 Da, Solarbio) overnight. Then, the sample was lyophilized to get powdery PCD.

### Preparation of ROS-triggered NPs

The drug-loaded NPs were prepared using a nanoprecipitation method. In brief, 10 mg of PCD powder and 1 mg of RAP were dissolved in 1 ml of formamide and methanol (1:1, v/v), the mixture was then added dropwise into 3 ml of ultra-pure water under magnetic stirring. Next, the solution was dialyzed (MWCO 3500 Da, Solarbio) against deionized water to remove unloaded drugs and organic reagents. The RAP-loaded NPs was denoted as RNPs. The bare NPs was denoted as PCD NPs. To prepare the fluorescently labeled NPs, 0.1 wt% DiD (excitation = 644 nm, emission= 665 nm) was loaded into PCD (DiD NPs).

### Preparation of MM-derived vesicles

RAW 264.7 cells were cultured in DMEM medium and supplemented with 10% (v/v) FBS at 37°C in 5% CO_2_ atmosphere. The cells which grew to 90% fusion were washed with icy PBS (pH 7.4) for three times, then collected by cell scraper and suspended in 1 ml of icy PBS. The collected cell suspension was centrifuged at 100 rcf for 5 min to get the cells. Cells were then dispersed in the membrane protein extraction buffer solutions and incubated for 15 min on ice. Next, the suspension was homogenized for 30 times by a glass homogenizer. The cell homogenate was centrifuged at 800 rcf for 10  min at 4°C to remove the released organelles. Subsequently, the supernatant was collected and centrifuged at 8600 rcf for 30 min to obtain MM. In order to obtain nano-sized macrophage vesicles (MVs), the extracted MM were extruded 10 times through a 400 nm polycarbonate porous membrane using an Avestin mini extruder (Avestin, LF-1, Canada). The harvested MVs were stored in water at 4°C.

### Preparation of MM-camouflaged ROS-responsive NPs

RNPs and MVs were mixed at a membrane protein-to-polymer ratio of 1:1 (w/w), the mixture was then sonicated in a water bath ultrasound machine (FS30D, 42 kHz, 100 W) for 2 min. The mixture was extruded through a polycarbonate membrane mini-extruder (Avestin, LF-1, Canada) for 15 times. Finally, MM/RNPs were collected and stored at 4°C for future use.

### General characterization of NPs

The structure of PCD was characterized by ^1^H-nuclear magnetic resonance (^1^H-NMR) spectroscopy using a Bruker Avance spectrometer (AVII-400; Bruker, Karlsruhe, Germany) at 400 MHz, DMSO-*d_6_* and methanol-*d_4_* (1:1, v/v) was used as the solvent. The hydrodynamic diameters and zeta potentials were measured by a Malvern Zetasizer Nano ZS unit (Nano ZS 90, Malvern, UK). Transmission electron microscopy (TEM, LIBRA 200 FE, Zeiss, Germany) at 200 kV was used to determine the morphology of RNPs, MM/RNPs and degraded PCD NPs. To make the TEM sample, a drop of the NPs solution at a concentration of 150 µg/ml was deposited onto a copper mesh and stained with 1% phosphotungstic acid. To investigate the ROS responsiveness, PCD NPs were, respectively, incubated with different concentrations of H_2_O_2_, hydroxyl radical (•OH) and hypochlorite (ClO^−^) for 2 h, followed by the measurement of hydrodynamic sizes by DLS. Additionally, PCD NPs solutions were incubated with and without the addition of 1 mM H_2_O_2_. The color changes during incubation were documented by digital photos at different time intervals. The morphology of degraded NPs was examined by TEM.

### Protein detection

The proteins of macropahge cells (MCs), MVs and MM/RNPs were extracted by a protein extraction kit (Beyotime). For sodium dodecyl sulfate polyacrylamide gel electrophoresis (SDS-PAGE), the protein samples and marker were loaded on the SDS-PAGE gel and run at 90 V for 0.5 h and then run at 140 V for 1 h using a Bio-Rad electrophoresis system. Finally, the gel was stained with Coomassie brilliant blue and washed for 3 times before visualization.

For western blotting (WB) analysis, proteins were transferred onto polyvinylidene difluoride membranes (PVDF membrane, Millipore, USA) from SDS-PAGE gel. Next, the PVDF membranes were blocked in 5% milk for 1 h before incubating with primary antibody (anti-integrin α4 (8440S, CST); anti-integrin β1 (34971, CST); anti-CD47 antibody (ab175388, Abcam)) at 4°C overnight. Afterwards, the membranes were washed with PBST and incubated with HRP-conjugated secondary antibody (Beyotime, Jiangsu, China) for 2 h. Finally, the membranes were washed with PBST again and observed by ChemiDoc-XRS imaging system (Bio-Rad, USA).

### Drug loading and *in vitro* drug release study

The drug loading efficiency (DLE) and drug encapsulation efficiency (DEE) were measured using UV/Vis Spectrophotometer (DU730, Beckman Coulter) at a 292-nm wavelength and calculated according to the standard curve and equations (1) and (2) to get the final result.

(1) $$\text{DLE}=\frac{\text{Mass}\;\text{of}\;\text{RAP}}{\text{Mass}\;\text{of}\;\text{PCD}+\text{Mass}\;\text{of}\;\text{RAP}}\times 100\%$$

(2) $$\text{DEE}=\frac{\text{Mass}\;\text{of}\;\text{RAP}}{\text{Mass}\;\text{of}\;\text{the}\;\text{added}\;\text{RAP}}\times 100\%$$

Drug release from RNPs and MM/RNPs were characterized using a dialysis method. Briefly, 1 ml of NPs solutions (500 µg/ml) was put into a dialysis bag (MWCO 3500 Da, Solarbio) and incubated in 10 ml of release medium (PBS: DMSO = 8:2, v/v) with or without H_2_O_2_, at predetermined time points, 1 ml of fresh medium was replenished after 1 ml of the original release medium was withdrawn for UV/Vis analysis. The cumulative amount of released RAP was measured using UV/Vis Spectrophotometer (DU730, Beckman Coulter) at a 280 nm wavelength and calculated based on the standard curve and equation (3).

(3) $$\text{Cumulative}\;\text{drug}\;\text{release}=\frac{M_{t}}{M_{0}}\times 100\%$$

where M_*t*_ and M_0_ represent the amount of drug released at time *t* and the initial amount of drug in the NPs, respectively.

### *In vitro* cellular uptake

*In vitro* cellular uptake of DiD NPs and MM/DiD NPs is conducted in macrophages. For confocal laser scanning microscopy (CLSM) study, macrophages were seeded on cell slides in 24-well plate with a density of 1 × 10^5^ cells/well. After 12 h incubation in 37°C incubator containing 5% CO_2_, 50 μl (2.5 mg/ml) of DiD NPs or MM/DiD NPs solutions were added into each well, then the cells were incubated with the solutions for 2 and 4 h, respectively. The medium was discarded and the cells were washed twice with PBS. Subsequently, 4% paraformaldehyde was added into each well and continue incubation for 30 min. After washing with PBS for three times, DAPI staining was performed for 30 min. Finally, after washing with PBS for three times again, the cell slides were taken out for CLSM (Leica, Germany) observation.

Quantification of NPs uptake in macrophages was measured by flow cytometry (FCM) (BD, USA). Macrophages were seeded in 12-well plate with a density of 1 × 10^6^ cells/well. After 12 h incubation in 37°C incubator containing 5% CO_2_, 150 μL (2.5 mg/ml) of DiD NPs or MM/DiD NPs solutions were added into each well, then the cells were incubated with the solutions for 2 and 4 h, respectively. After incubation, the cells were washed for three times with PBS and digested with trypsin for collection. Then, the cells were washed with PBS twice before centrifuge at 100 rcf for 5 min. Lastly, the cells were resuspended in 1 ml of PBS. In this experiment, DiD was used to replace RAP when preparing the fluorescence-labelled NPs. Three replicates were adopted for each group and 10 000 cells were analyzed in each sample. When setting the gate, the threshold value was set as 10^3^ to exclude cell debris.

### Cytotoxicity

Human umbilical vein endothelial cells (HUVECs) were seeded in 96-well plate at a density of 1 × 10^4^ cell/well in 100 μl of RPMI 1640 medium containing 10% (v/v) FBS. After incubation for 12 h, the cells were washed with sterilized PBS before the addition of fresh medium containing different concentrations of PCD NPs and MM/PCD NPs (10, 50, 100, 150, 200 μg/ml). After incubation for 24 h, the cell viability was quantified by MTS assay.

### Hemolysis assay

Four milliliters of rabbit blood (rabbit blood:anticoagulant = 9:1, v/v) was mixed with 5 ml of normal saline for dilution. Then, 40 μl of diluted rabbit blood was added to RNPs or MM/RNPs solutions (2 ml, 1 mg/ml). Normal saline was used as negative control, distilled water as positive control. After incubation in a 37°C water bath for 1 h, the samples were centrifuged at 800 rcf for 5 min. Afterwards, the supernatant of all samples were taken and measured the absorbance at 545 nm by UV-Vis (DU730, Beckman Coulter), then the hemolysis rate was calculated according to the following formula.

(4) $$\text{Hemolysis}\;\text{Rate}=\frac{A_{s}{-A}_{n}}{A_{p}{-A}_{n}}\times 100\%$$

where A_*s*_, A_*p*_ and A_*n*_ represent the absorbance of the sample, the positive control and the negative control, respectively.

### Inhibition of *in vitro* cell proliferation

The anti-proliferation effects of different formulations were evaluated with RAW 264.7 and smooth muscle cells. Cells were seeded in 96-well plate at a density of 0.5 × 10^4^ cell/well in 100 μl of DMEM medium containing 10% (v/v) FBS. After incubation for 12 h, the medium was replaced with fresh growth medium containing free RAP, RNPs or MM/RNPs at different RAP concentration (20 or 40 μg/ml). After incubation for 24 h, the cell viability was quantified by MTS assay.

### *In vitro* targeting ability of MM/RNPs

HUVECs were seeded on cell slides in 24-well plate with a density of 1 × 10^5^ cells/well. After 12 h incubation, cells were incubated with 50 ng/ml of TNF-α for 24 h to activate HUVECs. Then 40 µl of DiD NPs and MM/DiD NPs were added to non-activated or activated HUVECs. After 2 h incubation, the medium was discarded and the cells were washed twice with PBS. Subsequently, 4% paraformaldehyde was added into each well and continue incubation for 30 min. After washing with PBS for three times, DAPI staining was performed for 30 min. Then, the cells were taken out for CLSM observation.

### *In vivo* toxicity evaluation

Wild-type zebrafish were maintained in a 14 h:10 h light−dark cycle. Embryos were obtained via natural mating. After collection, the embryos were maintained in PTU solution (0.003%) at 28.0 ± 0.5°C. At 12 h postfertilization (hpf), the healthy embryos were selected for *in vivo* toxicity study. The selected embryos were randomly divided into 10 groups and placed in a 12-well plate, each group contains 10 embryos. Two milliliters of medium solution containing 0, 10, 100, 250 and 500 µg/ml of PCD NPs and MM/PCD NPs were added into different wells. The viability and developmental toxicity of the embryos were documented photographically at 12, 24, 48 and 72 hpf, respectively.

### Statistical analysis

All data were reported as mean ± SD. GraphPad Prism Version 6.0 software (GraphPad, USA) was used for the statistical analysis. One-way analysis of variance (ANOVA) was used to reveal differences among the groups. Unpaired *t*-test (two tails) was utilized to annotate statistical significance between two groups. The difference significance levels were set at *P <* *0.05, **P <* *0.01, ***P <* *0.001, ****P < 0.0001.

## Results and discussion

### Preparation and characterization of NPs

**Figure 2.**
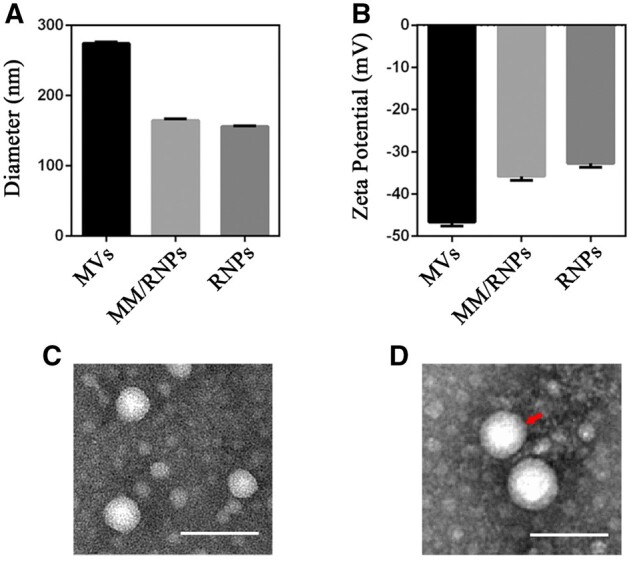
Sizes (**A**) and zeta potentials (**B**) of MVs, MM/RNPs and RNPs (*n *=* *3). TEM images of RNPs (**C**) and MM/RNPs (**D**); scale bar = 100 nm.

PCD copolymer was synthesized through the boronic esters chemically conjugating onto hydroxyl groups of dextran, confirmed by ^1^H-NMR. According to ^1^H-NMR result (Supplementary Fig. S1), the graft ratio of dextran coupled to boronic ester was 57.47%. RNPs were prepared *via* the nanoprecipitation methods, and MM/RNPs were fabricated by coating MM onto the surface of RNPs *via* a direct co-extrusion method. Compared with RNPs with an average diameter of 156.2 ± 0.76 nm and zeta potential of -32.7 ± 0.9 mV, the average hydrodynamic diameter of MM/RNPs slightly increased to 164.7 ± 2.1 nm (Fig. 2A), and the zeta potential increased to -35.8 ± 1.04 mV (Fig. 2B) due to MVs showing a higher negative surface charge of -46.6 ± 0.9 mV. The increase of the particle size and changes in zeta potential of MM/RNPs were attributed to the coating of MM, showing an evidence of successful fabrication of MM/RNPs. The morphology of RNPs and MM/RNPs were characterized by TEM. TEM images (Fig. 2C and D) showed that both RNPs and MM/RNPs exhibited a spherical structure. Especially, the cell membrane coated structure of MM/RNPs was clearly observed in the TEM image (Fig. 2D).

**Figure 3.**
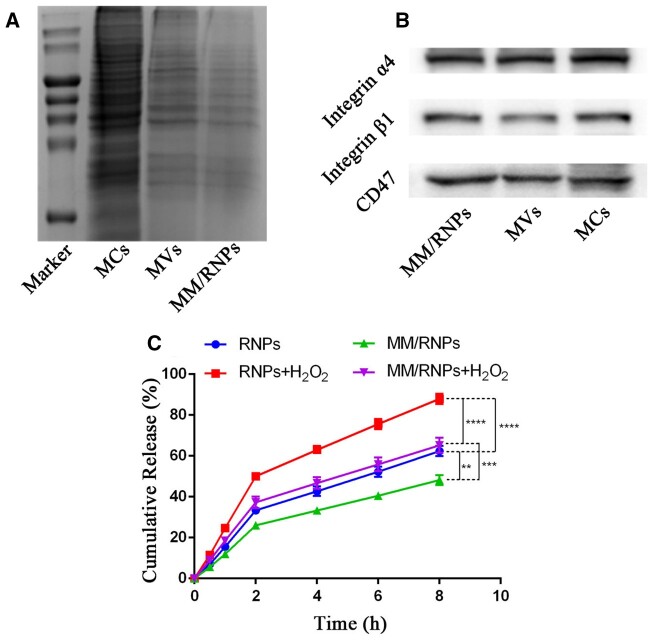
Characteristic protein bands of MCs, MVs and MM/RNPs resolved by SDS-PAGE (**A**) and WB analysis (**B**). *in vitro* drug release profiles (**C**) of RAP from RNPs and MM/RNPs with or without 1 mM H_2_O_2_. Data are presented as mean ± SD (**p<0.01, ***p<0.001, ****p<0.0001) (n = 3).

The proteins of cell membranes coated on the NPs play an important role in the biological function of the biomimetic NPs [34]. To investigate the retention of the functional membrane proteins, the membrane protein profiles of MCs, MVs and MM/RNPs were detected by SDS-PAGE. The SDS-PAGE result showed that MVs retained most of the protein compositions of MCs, and MM/RNPs had the similar protein profiles to MVs (Fig. 3A), which indicated that the membrane proteins were mostly retained during the fabrication process of MM/RNPs.

According to the previous reports [35, 36], the main functional proteins, CD47 and integrin α4β1, were generally expressed on the surface membrane of macrophage cells. CD47, an immunomodulatory protein, is capable of inhibiting macrophage phagocytosis through interaction with the signal regulatory protein-α (SIRP-α) receptor. Integrin α4β1 can specifically recognize and bind to vascular cell adhesion molecule-1 (VCAM-1), which is highly expressed on inflammatory endothelial cells of AS lesion site. Therefore, WB analysis was carried out to characterize these main functional proteins. The protein bands of CD 47, integrin α4 and integrin β1 in MCs, MVs and MM/RNPs were all imaged, suggested that MM/RNPs inherited the functional proteins from macrophage (Fig. 3B). In general, these results suggested that not only MVs were successfully translocated onto the surface of RNPs, but also MM/RNPs retained the main functional proteins to develop stealthy and targeted effects for potential advanced drug delivery.

Study has shown that aryl boronic esters are highly responsive to H_2_O_2_ [37]. More importantly, H_2_O_2_ is a key component of ROS and plays an important role in the pathogenesis of chronic inflammation. Therefore, the H_2_O_2_ responsiveness of PCD NPs was firstly investigated. Hydrolysis of PCD NPs was examined in aqueous solution with 1 mM H_2_O_2_. The test was conducted and observed over time with and without H_2_O_2_ at 37°C. In the presence of H_2_O_2_, the transparency of PCD NPs solution changed from opalescence to clear within 30 min. However, in the absence of H_2_O_2_, there was no significant difference in the transparency even after 2 h (Supplementary Fig. S2)_._ The transparency change could be resulted from cleavage of the boronic ester bonds in PCD triggered by H_2_O_2_. Additionally, the particle size of PCD NPs was measured by DLS after incubation with different concentrations of H_2_O_2_ for 2 h. A multimodal size distribution appeared after PCD NPs were stimulated by H_2_O_2_ (Supplementary Fig. S3). When the concentration of H_2_O_2_ increased, the particle size of PCD NPs increased correspondingly. The morphology of the degraded NPs was further observed by TEM, exhibiting significantly degradation into irregular structure after incubated with 1 mM H_2_O_2_ for 30 min (Supplementary Fig. S4).

In order to investigate the sensitivity of PCD NPs to different ROS, the hydrolysis of PCD NPs was studied in aqueous solutions containing different ROS reagents. In this study, two types of ROS, hydroxyl radical (•OH) and hypochlorite (ClO^−^), were chosen for this experiment according to the previous report [38], and the size changes of PCD NPs were detected by DLS. As shown in Supplementary Fig. S5, the particle size of PCD NPs generally increased after incubation with higher ROS concentration. In addition, compared with a monodisperse size distribution in the control group (PCD solution without ROS stimulus), the particle size of PCD NPs stimulated by ROS showed a multimodal distribution, indicating that the NPs were unstable. The increase of particle size of PCD NPs stimulated by ROS may be attributed to the loose structure of the degraded NPs. These results indicated that PCD NPs can respond to a variety of ROS stimuli to degrade and subsequently accelerate drug release in the ROS-rich microenvironment.

Subsequently, the ROS-responsive drug release behavior of NPs was investigated in buffers with or without H_2_O_2_. The DEE and DLE of RNPs were, respectively, 65.17 and 8.5%, measured by UV-Vis spectrophotometer (Supplementary Table S1). After 8 h incubation with H_2_O_2_, around 87.95% of RAP was cumulatively released from RNPs, in contrast of only 62.42% of that from RNPs without H_2_O_2_. Similarly, the cumulative release amount of MM/RNPs were 48.22 and 65.2% after 8 h incubation without and with H_2_O_2_, respectively (Fig. 3C). The results showed that the presence of ROS stimulus could accelerate the RAP release from NPs. In addition, the cumulative release of RAP from MM/RNPs was lower than RNPs, no matter with or without H_2_O_2_, which could be attributed to the MM coating acting as a barrier to slow drug release from RNPs. The results suggested that the NPs can respond to ROS stimuli for controlling drug release. Therefore, the ROS-responsive MM/RNPs have a great potential to efficiently deliver and control drug release into the pathological ROS-rich microenvironment of atherosclerotic plaques.

### *In vitro* uptake of MM/RNPs by macrophages

**Figure 4.**
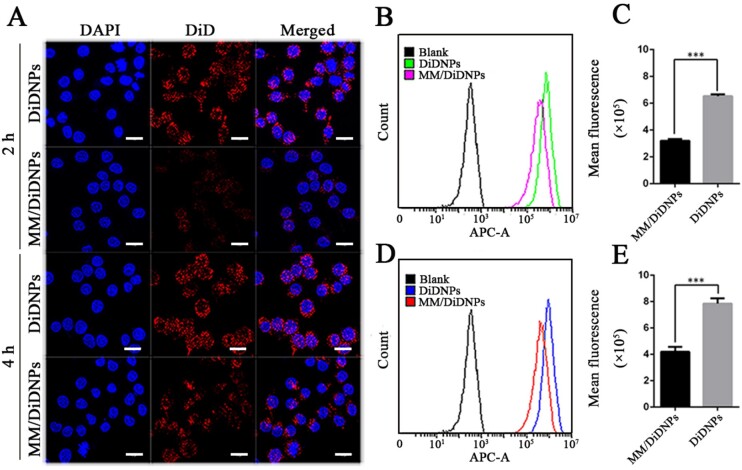
*In vitro* macrophage uptake. CLSM images (**A**) of RAW264.7 cells after incubation with DiD NPs and MM/DiD NP; scale bar = 15 µm. FCM analysis of RAW264.7 cells after incubation with DiD NPs and MM/DiD NP for 2 h (**B**, **C**) and 4 h (**D**, **E**), respectively. Data are presented as mean ± SD (***P <* *0.001) (*n *=* *3).

MM-coated NPs were found to possess the capability of protecting the NPs from the elimination of immune system [39]. The ability of MM-coated NPs to inhibit macrophage uptake was studied by CLSM. Macrophages were treated with MM/DiD NPs and DiD NPs for 2 and 4 h, respectively. The uptake behavior of MM/DiD NPs and DiD NPs by macrophages exhibited a time-dependent manner. At the same time point, the cells incubated with DiD NPs showed significantly increased fluorescence intensity comparing to MM/DiD NPs (Fig. 4A). Furthermore, FCM analysis was used to quantify the uptake of NPs by macrophages. Macrophages incubated with MM/DiD NPs showed nearly half fluorescence intensity than that of DiD NPs after 2 and 4 h incubation (Fig. 4B−E), which may be attributed to CD47 proteins on the surface of MM/DiD NPs reducing the uptake of MM-coated NPs by macropahges. Taken together, NPs coated with MM could effectively evade the clearance of mononuclear phagocyte system, having desirable potential to prolong the blood circulation time.

### *In vitro* biocompatibility and therapeutic efficacy

**Figure 5.**
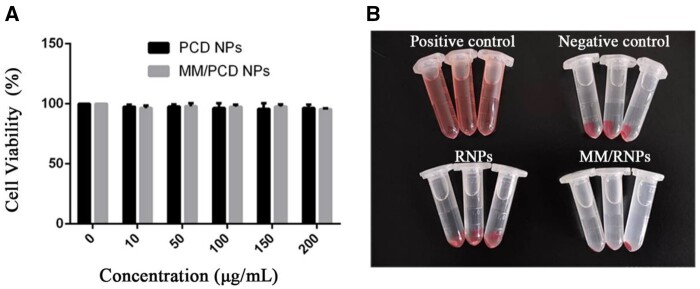
*In vitro* biocompatibility of NPs. *In vitro* cytotoxicity (**A**) of PCD NPs and MM/PCD NPs on HUVECs at different concentrations. Digital photo (**B**) of hemolysis test.

The *in vitro* cytotoxicity study was evaluated in HUVECs by MTS assay. After incubation for 24 h, the viability of HUVECs treated with blank NPs and MM-coated NPs had no significant difference when compared with the control group, even at the high concentration of 200 μg/ml (Fig. 5A). This result suggested that both PCD NPs and MM-coated NPs had good cytocompatibility.

Moreover, nanomedicines were commonly administrated through intravenous injection. The blood compatibility of nanomedicines is an important safety index [40]. Herein, the hemolysis test was carried out to evaluate the blood biocompatibility of MM/RNPs. Same with the negative control group, the dilute blood incubated with RNPs and MM/RNPs presented as clear solution after centrifugation (Fig. 5B). In addition, the hemolytic ratio of RNPs and MM/RNPs were further calculated as 3.7 and 4.6% (Supplementary Table S2), respectively, indicated that both RNPs and MM/RNPs are an hemolytic for safe application *in vivo*.

**Figure 6.**
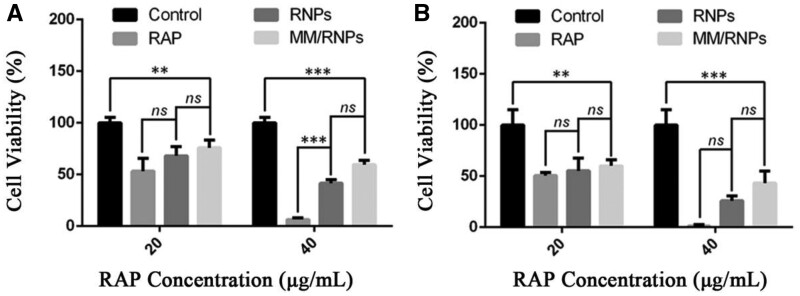
*In vitro* anti-proliferation ability evaluations of NPs. Cell viability of RAW 264.7 cells (**A**) treated with RAP, RNPs and MM/RNPs. Cell viability of smooth muscle cells (**B**) treated with RAP, RNPs and MM/RNPs. Data are presented as mean ± SD (**P <* *0.01, ***P <* *0.001 and ns, no significance) (*n *=* *3).

The abnormal proliferation of macrophages and smooth muscle cells in AS lesions can promote the development of AS [41]. Therefore, the anti-proliferation ability of RAP-loaded NPs were evaluated in macrophages and smooth muscle cells by MTS assay. The free RAP, RNPs and MM/RNPs, at the RAP concentration of 20 and 40 µg/ml, were added to treat both of macrophages and smooth muscle cells for 24 h, respectively. All the formulations could inhibit macrophages and smooth muscle cells in a dose-dependent manner (Fig. 6). Free RAP, RNPs and MM/RNPs could significantly inhibit the proliferation of macrophages and smooth muscle cells at 20 µg/ml of RAP. While free RAP showed higher anti-proliferation effects on cells, RNPs and MM/RNPs also exhibited favorable anti-proliferation effects when compared with the same dose of free RAP. The stronger anti-proliferative activity of free RAP could be ascribed to the controllable RAP release from RNPs and MM/RNPs. Together, these findings indicated that the nanotherapies exhibited potent anti-proliferation ability to macrophages and smooth muscle cells.

### Evaluation of *in vitro* NP targeting ability and *in vivo* toxicity

**Figure 7.**
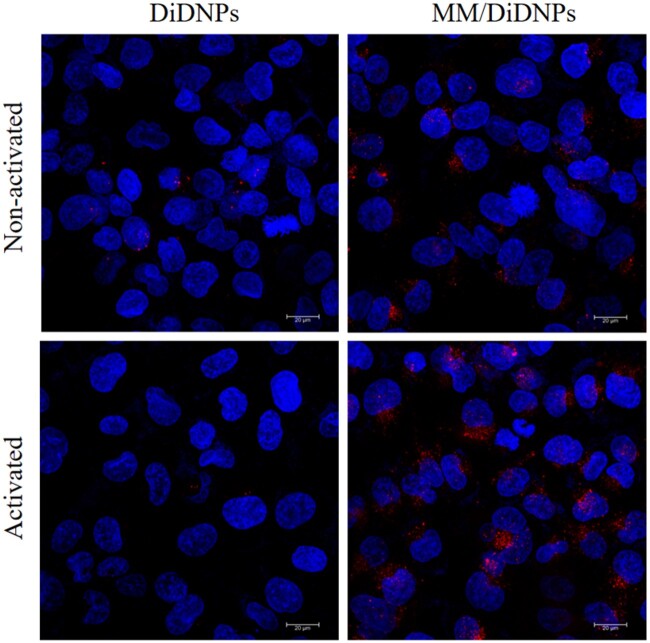
*In vitro* targeting of MM/DiD NPs toward inflammatory endothelial cells; scale bar = 20 µm.

One of the key events of atherogenesis is the activation of ECs, the inflammatory ECs express a series of adhesion molecules that can recruit macrophages to the atherosclerotic plaques. So, the targeting ability of MM-coated NPs to inflammatory ECs was investigated *in vitro.* Normal HUVECs and inflammatory HUVECs induced by tumor necrosis factor-α (TNF-α) were incubated with MM/DiD NPs, or DiD NPs for 2 h. The CLSM images showed that a large amount of MM/DiD NPs accumulated in inflammatory HUVECs, whereas normal HUVECs treated with MM/DiD NPs exhibited less NPs accumulation. Meanwhile, only a little amount of NPs was observed in both normal and induced HUVECs incubated with bare NPs (Fig. 7). These results suggested that coating with MM enabled the NPs to actively target inflammatory ECs *in vitro*, which could be ascribed to the immune recognition of MM receptors with the adhesion molecules on inflammatory HUVECs.

**Figure 8.**
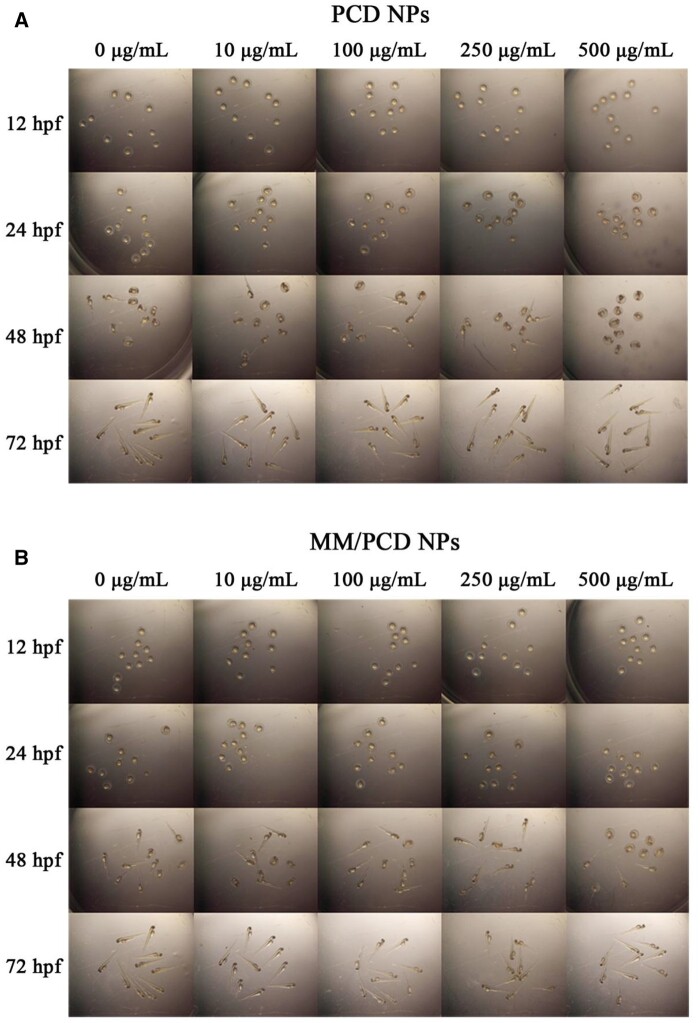
*In viv*o toxicity study of PCD NPs and MM/PCD NPs on zebrafish embryos. The images of zebrafish embryos incubated with 0, 10, 100, 250 and 500 µg/ml of PCD NPs (**A**) and MM/PCD NPs (**B**) solution at 12, 24, 48 and 72 hpf.

To confirm the *in vivo* biocompatibility of PCD NPs and MM/PCD NPs, zebrafish was chosen as an animal model to examine the developmental toxicity of NPs. At 12 hpf, zebrafish embryos were selected and incubated with PCD NPs and MM/PCD NPs solutions at various concentrations (0, 10, 100, 250 and 500 µg/ml) and observed at 12, 24, 48 and 72 hpf by a stereomicroscope. Zebrafish treated with PCD NPs and MM/PCD NPs showed no death and no malformation at the developmental stage of embryos (Fig. 8), even at the highest concentration of 500 µg/ml, which suggested the good biocompatibility of PCD NPs and MM/PCD NPs *in vivo*.

## Conclusion

In this study, we fabricated the biomimetic ROS-responsive NPs for the targeted treatment of AS, in which RNPs served as the cores, followed by surface-coating with MM. The biomimetic ROS-responsive MM/RNPs have the controllable drug release behavior in response to H_2_O_2_ stimulus. In addition, the main functional proteins of natural MM are well retained on MM/RNPs through the coating process. Because of the biomimetic camouflage on surface, MM/RNPs can effectively inhibit the uptake of macrophages and selectively target to inflammatory endothelial cells *in vitro*. Meanwhile, the *in vitro* anti-proliferation experiments demonstrated that MM/RNPs can effectively inhibit the proliferation of macrophages and smooth muscle cells. Finally, preliminary *in vitro* hemolysis, cytotoxicity and *in vivo* developmental safety evaluations showed their favorable biocompatibility. Therefore, this biomimetic ROS-responsive MM/RNPs may represent a potential DDS to effectively treat AS.

## Supplementary data

Supplementary data are available at *REGBIO* online.

## Supplementary Material

rbab033_Supplementary_Data
