# Supplementary material for: ROS-responsive biomimetic nanoparticles for potential application in targeted anti-atherosclerosis
Source: Regen Biomater. 2021 Jul 18;8(4):rbab033. doi: 10.1093/rb/rbab033 (PMC8286794; doi:10.1093/rb/rbab033)
Supplement: rbab033_Supplementary_Data [file rbab033_supplementary_data.docx]

Supplementary Material

**ROS-responsive biomimetic nanoparticles for potential application in targeted anti-atherosclerosis**

Dan Tang^1‡^, Yi Wang^1,2‡^, Andy wijaya^1^, Boyan Liu^1^, Ali Maruf^1^, Jinxuan Wang^1^, Jianxiong Xu^1^, Xiaoling Liao^2^, Wei Wu^1*^, Guixue Wang^1^^*^

^1^Key Laboratory for Biorheological Science and Technology of Ministry of Education, State and Local Joint Engineering Laboratory for Vascular Implants, Bioengineering College of Chongqing University, Chongqing, 400030, China

^2^Chongqing Key Laboratory of Nano/Micro Composite Material and Device, School of Metallurgy and Materials Engineering, Chongqing University of Science and Technology, Chongqing, 401331, China

^‡^These authors contributed equally to this work.

*Corresponding authors: wanggx@cqu.edu.cn (Guixue Wang); david2015@cqu.edu.cn (Wei Wu)

**
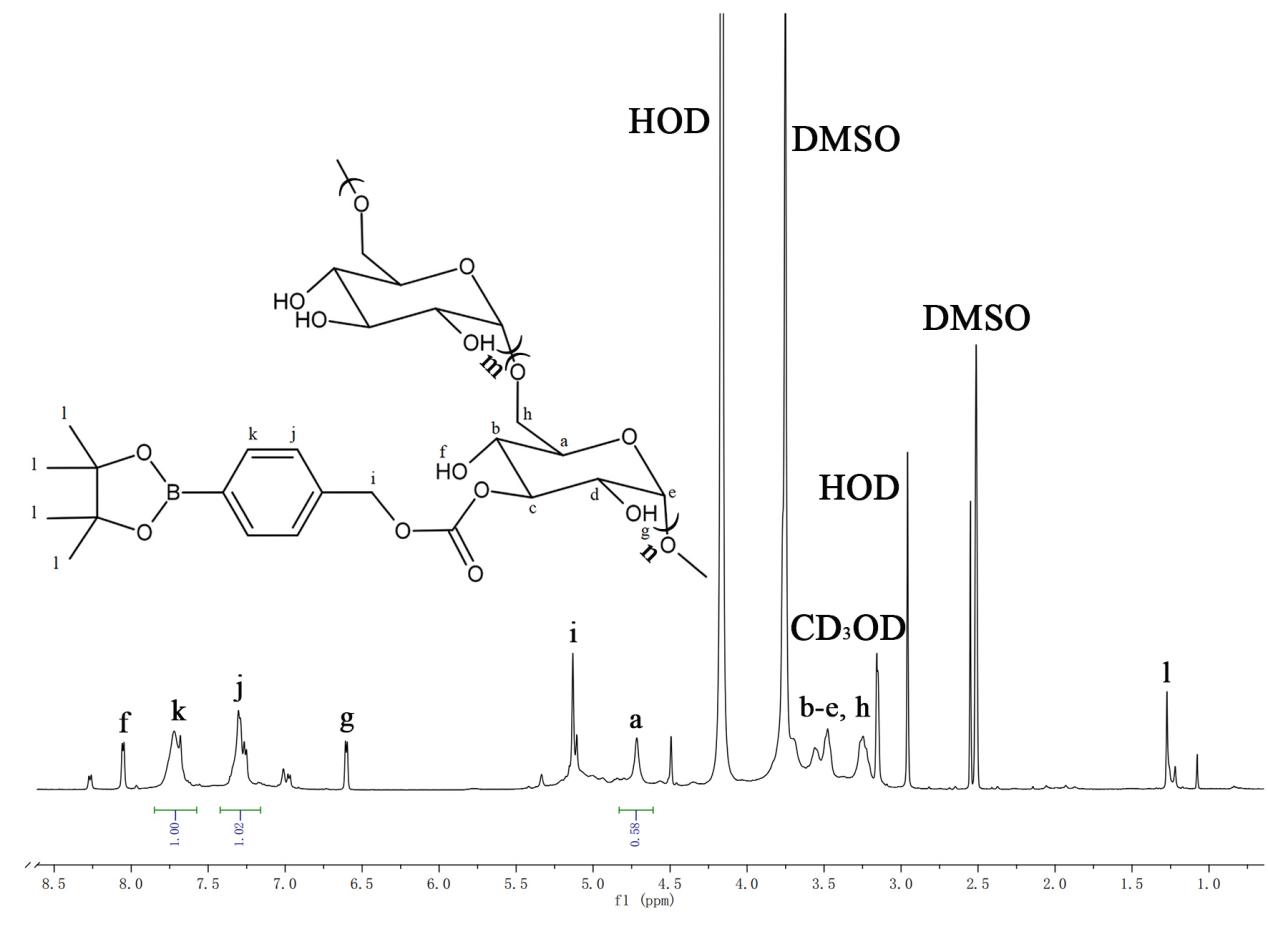
**

**Figure S1.** ^1^H-NMR spectrum of PCD in DMSO-*d_6_* and methanol-*d_4_*.


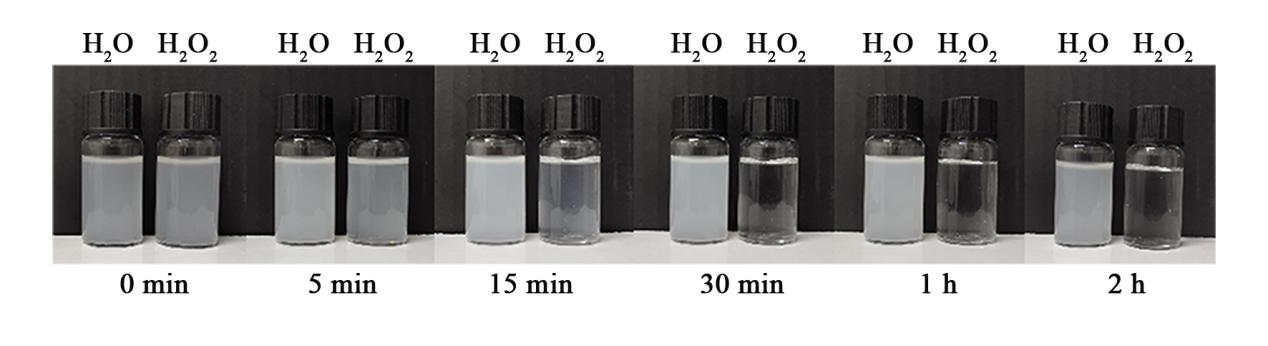


**Figure S2.** Digital photo of the time-dependent degradation of PCD NPs in 1 mM H_2_O_2_.


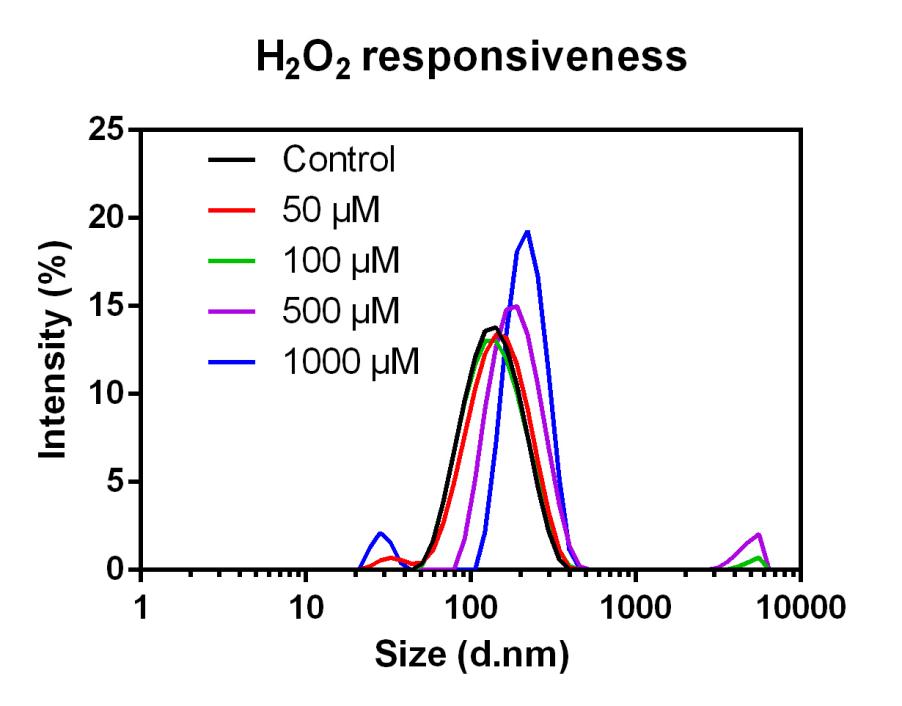


**Figure S3.** Size changes of PCD NPs after incubating with different concentrations of H_2_O_2_ for 2 h.


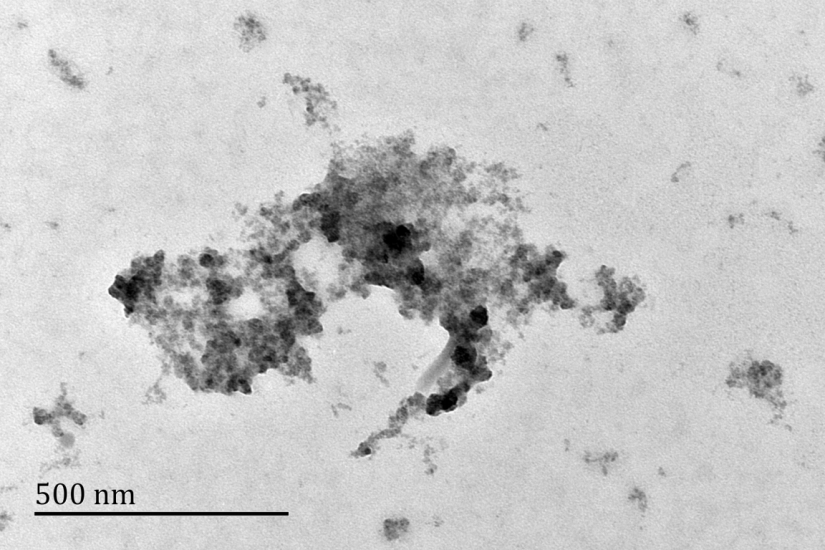


**Figure S4.** TEM image shows the degradation of PCD NPs incubated with 1mM H_2_O_2_ for 30 min.


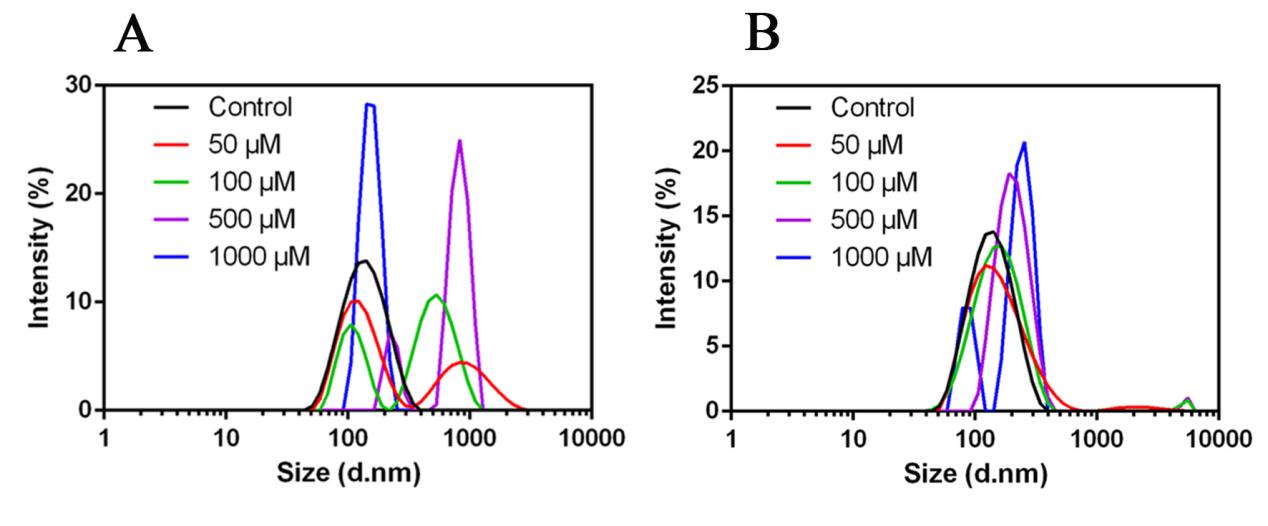


**Figure S5.** ROS-triggered size changes of PCD NPs incubated with different concentrations of •OH (A) and ClO^−^ (B) for 2 h.

**Table S1.** Drug loading and encapsulation efficiency of RNPs (*n* = 3).

| Sample | μg/mg | Drug Loading Efficiency (%) | Drug Encapsulating  Efficiency (%) |
| --- | --- | --- | --- |
| RNPs | 85 | 8.5 | 65.17 |

**Table S2**. Hemolysis ratio of RNPs and MM/RNPs (*n* = 3).

| Name | 1 | 2 | 3 | Average | Hemolysis Rate |
| --- | --- | --- | --- | --- | --- |
| RNPs | 0.0493 | 0.0507 | 0.0579 | 0.0526±0.0046 | 3.7% |
| MM/RNPs | 0.1846 | 0.196 | 0.2347 | 0.2051±0.0263 | 4.6% |
| Negative control | 0.0509 | 0.0331 | 0.0321 | 0.0387±0.0106 |  |
| Positive control | 1.0698 | 1.1159 | 1.0583 | 1.0813±0.0305 |  |
| MVs | 0.1179 | 0.1183 | 0.1177 | 0.118±0.0003 |  |
